# Complete Genome Sequences of Two Prolific Biofilm-Forming *Staphylococcus aureus* Isolates Belonging to USA300 and EMRSA-15 Clonal Lineages

**DOI:** 10.1128/genomeA.00610-14

**Published:** 2014-06-26

**Authors:** J. S. Sabirova, B. B. Xavier, J.-P. Hernalsteens, H. De Greve, M. Ieven, H. Goossens, S. Malhotra-Kumar

**Affiliations:** a Department of Medical Microbiology, Vaccine & Infectious Disease Institute, Universiteit Antwerpen, Antwerp, Belgium; b Viral Genetics Research Group, Vrije Universiteit Brussel, Brussels, Belgium; c Structural Biology Brussels, Vrije Universiteit Brussel, Brussels, Belgium; d Structural and Molecular Microbiology, Structural Biology Research Center, VIB, Brussels, Belgium

## Abstract

Methicillin-resistant *Staphylococcus aureus* (MRSA) causes serious infections that are even more difficult to treat when associated with a biofilm phenotype that facilitates evasion of the host immune system and antibiotics. As a first step toward understanding the mechanisms underlying biofilm formation, we sequenced the genomes of two prolific biofilm-forming strains belonging to the two most important globally disseminated clonal lineages, USA300 and EMRSA-15.

## GENOME ANNOUNCEMENT

Methicillin-resistant *Staphylococcus aureus* (MRSA) represents a major cause of hospital- and community-acquired infections ranging from minor skin infections to life-threatening diseases such as pneumonia, meningitis, osteomyelitis, endocarditis, and septicemia, as well as infections associated with medical implants and postsurgical wound infections. Prolonged persistence of MRSA infections is at least partly linked to the formation of biofilms *in vivo* (1), with biofilm-linked resistance to antibiotics posing a major problem to clinicians in the treatment of MRSA infections (2). Two of the most successful MRSA clones that have disseminated globally are the staphylococcal cassette chromosome *mec* type IV (SCC*mec* IV)-harboring USA300 and EMRSA-15 (3–5). We have recently identified two *S. aureus* strains, UAS391 and H-EMRSA-15, each belonging to one of these two clonal lineages as prolific biofilm producers (E. Vanhommerig, P. Moons, D. Pirici, C. Lammens, J.-P. Hernalsteens, H. De Greve, S. Kumar-Singh, H. Goossens, S. Malhotra-Kumar, submitted for publication). The UAS391 and H-EMRSA-15 strains belong to multilocus sequence types (ST) 80 and 22, respectively, with the former also harboring the *pvl* gene and an arginine catabolic mobile element (ACME) I element. This study reports the whole-genome sequencing of UAS391 and H-EMRSA-15, which is a prerequisite to understanding the molecular mechanisms underlining their pronounced clinical and biological phenotypes.

Whole-genome (optical) maps of UAS391 and H-EMRSA-15 were obtained using the Argus Optical Mapping System (OpGen, Gaithersburg, MD). Comparison with *in silico* whole-genome maps generated from publicly available *S. aureus* whole-genome sequences identified two strains as the closest homologs. UAS391 was found to have a whole-genome map identical to that of *S. aureus* USA300_TCH1516 (GenBank accession no. NC_010079), whereas the map of H-EMRSA-15 showed 99.4% similarity to that of *S. aureus* HO 5096 0412 (GenBank accession no. HE681097), as H-EMRSA-15 possesses an additional genomic insertion (14,218-bp island) between nucleotide positions 409805 and 409733. The complete sequences of UAS391 and H-EMRSA-15 were then determined on an Illumina HiSeq2000 platform (GIGA, University of Liège, Belgium). Sequence data of UAS391 and H-EMRSA-15 were *de novo* assembled using Velvet and CLC Genomics Workbench 6.5.1 (CLC, Inc., Aarhus, Denmark). Assembled contigs were ordered against the UAS391 and H-EMRSA-15 whole-genome maps using MapSolver software (OpGen, Gaithersburg). *De novo* assembled sequences were further corroborated by reference assembly using genome sequences of TCH1516 and HO 5096 0412 as corresponding templates. Genome analysis of UAS391 and H-EMRSA-15 is in progress to reveal the genetic and genomic basis of biofilm formation in these two major clonal lineages. We used the NCBI Prokaryotic Genome Automatic Annotation Pipeline (PGAAP) for function-based annotation (6). The chromosome size of UAS391 is 2,872,916 bp and of H-EMRSA-15 is 2,846,320 bp, and these contain 2,591 and 2,563 protein-coding genes, 59 and 57 tRNAs, and 5 and 6 rRNAs, respectively.

### Nucleotide sequence accession numbers.

The genome sequences for UAS391 and H-EMRSA-15 have been deposited at DDBJ/EMBL/GenBank under the accession numbers CP007690 and CP007659, respectively. The versions described in this paper are the first versions.
